# Longitudinal associations between incident lumbar spine MRI findings and chronic low back pain or radicular symptoms: retrospective analysis of data from the longitudinal assessment of imaging and disability of the back (LAIDBACK)

**DOI:** 10.1186/1471-2474-15-152

**Published:** 2014-05-13

**Authors:** Pradeep Suri, Edward J Boyko, Jack Goldberg, Christopher W Forsberg, Jeffrey G Jarvik

**Affiliations:** 1 Seattle Epidemiologic Research and Information Center, VA Puget Sound Health Care System, Seattle, WA USA; 2 Division of Rehabilitation Care Services, VA Puget Sound Health Care System, 1660 S. Columbian Way RCS-117 Seattle, WA, USA; 3 Department of Rehabilitation Medicine, University of Washington School of Medicine, Seattle, WA USA; 4 Department of Medicine, University of Washington School of Medicine, Seattle, WA USA; 5 Department of Epidemiology, University of Washington School of Public Health, Seattle, WA USA; 6 Department of Radiology, University of Washington School of Medicine, Seattle, WA USA; 7 Department of Neurological Surgery, University of Washington School of Medicine, Seattle, WA USA; 8 Department of Health Services, University of Washington School of Public Health, Seattle, WA USA

**Keywords:** Sciatica, Annular, Tear, Fissure, Herniation, Bulge, Impingement, Endplate, Modic, Stenosis, Extrusion, Spondylolisthesis, Facet, Arthritis, Zygapophyseal, Disc

## Abstract

**Background:**

There are few longitudinal cohort studies examining associations between incident MRI findings and incident spine-related symptom outcomes. Prior studies do not discriminate between the two distinct outcomes of low back pain (LBP) and radicular symptoms. To address this gap in the literature, we conducted a secondary analysis of existing data from the Longitudinal Assessment of Imaging and Disability of the Back (LAIDBACK). The purpose of this study was to examine the association of incident lumbar MRI findings with two specific spine-related symptom outcomes: 1) incident chronic bothersome LBP, and 2) incident radicular symptoms such as pain, weakness, or sensation alterations in the lower extremity.

**Methods:**

The original LAIDBACK study followed 123 participants without current LBP or sciatica, administering standardized MRI assessments of the lumbar spine at baseline and at 3-year follow-up, and collecting information on participant-reported spine-related symptoms and signs every 4 months for 3 years. These analyses examined bivariable and multivariable associations between incident MRI findings and symptom outcomes (LBP and radicular symptoms) using logistic regression.

**Results:**

Three-year cumulative incidence of new MRI findings ranged between 2 and 8%, depending on the finding. Incident annular fissures were associated with incident chronic LBP, after adjustment for prior back pain and depression (adjusted odds ratio [OR] 6.6; 95% confidence interval [CI] 1.2-36.9). All participants with incident disc extrusions (OR 5.4) and nerve root impingement (OR 4.1) reported incident radicular symptoms, although associations were not statistically significant. No other incident MRI findings showed large magnitude associations with symptoms.

**Conclusions:**

Even when applying more specific definitions for spine-related symptom outcomes, few MRI findings showed large magnitude associations with symptom outcomes. Although incident annular fissures, disc extrusions, and nerve root impingement were associated with incident symptom outcomes, the 3-year incidence of these MRI findings was extremely low, and did not explain the vast majority of incident symptom cases.

## Background

It is generally accepted that obtaining lumbar spine magnetic resonance imaging (MRI) for new low back pain (LBP) is of little value in making a diagnosis based on specific spinal pathoanatomic changes [1]. The high prevalence of many lumbar spine MRI findings in individuals without current LBP supports this view [2,3]. Many studies have examined cross-sectional associations between lumbar spine MRI findings and LBP, with most MRI findings demonstrating no significant associations with LBP, or associations of small magnitude [4,5]. However, few longitudinal studies have examined whether *incident* LBP is associated with *incident* lumbar spine MRI findings, compared to a known MRI baseline prior to the onset of pain. Longitudinal imaging studies of this type correspond to the clinical scenario where a patient with new LBP or sciatica develops a new finding on lumbar spine MRI, raising the question: “Is this new MRI finding the cause of symptoms in this patient?”. Such studies allow assessment of temporality and greater confidence in making causal inferences. In addition, when assessing the longitudinal effects of changes in a MRI finding on symptom outcomes, each participant effectively serves as their own control, reducing the effect of certain potential confounders that may differ among individuals but which do not vary over time for a participant [6].

**Figure 1:**
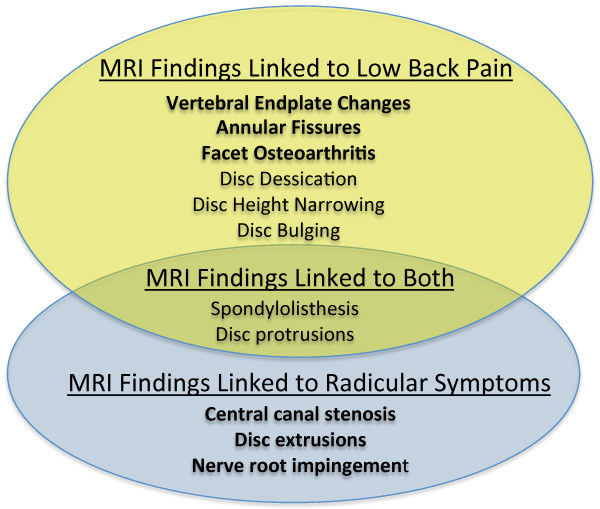
Conceptual links between MRI findings and spine-related symptoms*. *Primary MRI predictors of interest in bold.

There are several potential challenges involved in examining the association of lumbar spine MRI findings with LBP and other spine-related outcomes such as radicular pain (or ‘sciatica’). First, there is no consensus on the optimal case definition for LBP, and these may vary considerably in regards to pain persistence and severity [7]. Since almost all individuals experience acute LBP at some point in their lives, and most acute episodes resolve without major disability or work interference [8,9], LBP definitions emphasizing persistent and severe pain have been proposed as preferable outcome measures for LBP research [4,10]. Second, the experience of LBP is highly variable, with a clinical course ranging from complete symptom resolution to frequent recurrence [11-13]. This makes the assessment of incident LBP and related symptoms at a single follow-up timepoint unreliable for detecting whether incident symptoms have developed [12]. Third, many studies conducted previously do not clearly distinguish LBP from lower extremity radicular symptoms, such as sciatica [4]. This is in stark contrast to clinical practice, where spine clinicians expend much effort trying to distinguish whether symptom presentations predominantly involve LBP, or lower extremity neuropathic pain due to lumbosacral radicular syndrome or neurogenic claudication. Since certain MRI findings may be associated primarily with axial LBP (such as vertebral endplate (‘Modic’) changes, annular fissures, and disc degeneration) and other findings are associated with radicular symptoms or neurogenic claudication (such as spinal stenosis, disc extrusions, and nerve root impingement), the combining of LBP and lower extremity symptoms into composite definitions may obscure real associations that pertain to more specific symptom outcomes. Figure 1 depicts common lumbar spine MRI findings and the symptom outcomes (LBP vs. radicular symptoms) to which they are most closely linked conceptually, based on associations in the scientific literature or in actual clinical practice. For the purposes of this article, we will use the term ‘radicular symptoms’ to refer broadly to changes associated with lumbosacral radicular syndrome and/or neurogenic claudication, including radiating lower extremity pain (sciatica), sensation changes, and motor changes.

We conducted an examination of longitudinal associations between incident MRI findings and incident back-related symptoms and signs, accounting for these potential methodologic problems. This was a secondary analysis of existing data collected during the Longitudinal Assessment of Imaging and Disability of the Back (LAIDBACK), which was conducted between 1997 and 2005 in Seattle, Washington [14]. The purpose of this study was to examine the association of incident lumbar MRI findings with two specific symptom outcomes: 1) incident *chronic bothersome LBP*, and 2) incident *radicular symptoms* (i.e. pain, weakness, or sensation alterations in the lower extremity). This analytic approach is distinct from that taken in the original LAIDBACK longitudinal analysis [14], which combined LBP and radicular symptoms into a composite outcome, and did not account for the persistence and severity of LBP symptoms. Furthermore, we examined whether the effects of incident MRI findings varied according to the symptom outcome (chronic LBP vs. radicular symptoms).

## Methods

### Study participants

The LAIDBACK cohort included participants without current or recent LBP or sciatica, randomly sampled from amongst patients attending primary care, dental, and dermatology clinics at the Veterans Affairs (VA) Puget Sound Health Care System. Exclusion criteria included: LBP more than “mildly bothersome” in the last 4 months or Roland disability score >3; any sciatica in the last 4 months; prior acute back trauma or invasive spine procedures; and comorbidities limiting study participation [14,15]. Informed consent for participation in the study was obtained from the participants. All participants received lumbar spine MRI assessments at the time of enrollment; this secondary analysis included only the subset of LAIDBACK participants who also completed a second lumbar spine MRI assessment at 3-year follow-up. Participants reported on spine-related symptoms and signs every 4 months during this 3-year period [14,15]. The database used for this study is not freely accessible; permission to use the database was granted by Jerry Jarvik, MD, MPH (the senior author of this study). This work was approved by the Institutional Review Board of VA Puget Sound Health Care System.

### Longitudinal assessment of incident LBP and radicular symptoms

At the time of study enrollment, and every 4 months thereafter, participants rated the bothersomeness of four symptoms over the preceding 4 month period, using items from the Pain Frequency Index [14,16]: (1) low back or buttock pain; (2) leg pain (or sciatica); (3) numbness or tingling in the leg, foot, or groin; and (4) weakness in leg or foot. With regards to participant-reported leg pain/sciatica and lower extremity numbness, tingling, and weakness, no specific efforts were made to ascertain whether these reported symptoms were due to a spinal source, or another cause. Bothersomeness ratings ranged from 1 (‘not at all bothersome’) to 6 (‘extremely bothersome’) [14,15]. Investigators followed participants longitudinally with standardized telephone interviews every 4 months, until the 36-month follow-up, at which time the interviews were completed in person. At each interview, participants reported on symptoms over the past 4 months since the prior questionnaire [14,15]. We defined incident *chronic LBP* as low back or buttock pain that was ‘moderately’, ‘very’, or ‘extremely’ bothersome, at *two* or more timepoints over the 3-year follow-up. This definition includes both persistently bothersome and variably bothersome (recurrent) pain [13,17]. It is therefore distinct from the more commonly used outcome of ‘any LBP’, which would include brief episodes that resolve without meaningful functional consequences [8,9]. We defined incident *radicular symptoms* as any sciatica, lower extremity numbness or tingling, or lower extremity weakness, at *one* or more timepoints over the 3-year follow-up. This definition assumes any such radicular symptoms to be abnormal, irrespective of bothersomeness. These symptom definitions (chronic LBP and radicular symptoms) were specified prior to this analysis by the first author, who was blinded to the actual database, but was aware of the results of the original LAIDBACK publication [14]. These symptom definitions, however, were not originally planned as part of the original LAIDBACK study or the related applications for funding.

### Longitudinal assessment of the lumbar spine using MRI

We obtained sagittal and axial T1- and T2-weighted image sequences through each of the five lumbar intervertebral disc levels, using a Philips 1.5 Tesla MRI system. MRI scans were interpreted by one of two neuroradiologists (Jerry Jarvik, MD, MPH and David Haynor, MD, PhD), both senior members of the American Society of Neuroradiology and with extensive clinical and academic expertise in lumbar spine imaging. The neuroradiologists were blinded to all clinical information other than knowing that participants were asymptomatic at baseline. Readers graded MRI findings at the L1-S1 intervertebral disc levels, using standard definitions from the radiology literature (see Additional file 1). Interobserver reliability for MRI findings using the baseline MRI scans was calculated based on 10 scans (50 intervertebral discs) that were interpreted independently by both neuroradiologists. Interobserver reliability ranged from moderate (annular fissures: kappa = 0.54) to almost perfect (disc dessication: kappa = 0.84) [15]. After interobserver reliability was calculated, the neuroradiologists discussed cases where assessments of the baseline MRI findings differed, and tried to reach consensus, through an iterative process. Assessment of incident MRI findings was then done by direct comparison of the baseline and 3-year images together in pairs, by the same neuroradiologist, in order to minimize variability other than meaningful anatomic changes. Each neuroradiologist interpreted roughly half of the paired baseline and 3-year scans. Prior to analysis, MRI findings with stronger conceptual links with symptom outcomes were classified as ***primary MRI predictors of interest***, and grouped into two categories: 1) findings with links to LBP, and 2) findings with links to radicular symptoms; primary MRI predictors are listed in bold on Figure 1. Spondylolisthesis and disc protrusions were thought to have possible links to both chronic LBP and radicular symptoms, and were included in both categories. The decision to select specific MRI findings as primary MRI predictors of interest was based on these findings having either 1) larger magnitude associations in the literature (i.e. the association of vertebral endplate changes or annular fissures with LBP [4,18,19]), 2) stronger conceptual links based on clinical grounds (i.e. the association of central canal stenosis, disc extrusions, or nerve root impingement with radicular symptoms), or 3) findings which have been not been well studied previously (i.e. the association of facet joint hypertrophy with LBP [20-22]).

### Covariates

We examined covariates that are commonly accounted for in studies of LBP. These included age, sex, race, body mass index (kg/m^2^), current smoking status, depression, arthritis, and prior episodes of LBP and sciatica [14,15]. Our intent was not to examine all possible confounding factors, but rather to account for a limited list of factors that were thought to be of potential importance. Self-reported depression and arthritis were ascertained using items from the Self-Administered Comorbidity Questionnaire (SACQ), a reliable and valid comorbidity measure that is widely used in orthopedic research [23]. Self-report of ‘arthritis’ in this version of the SACQ does not attempt to distinguish osteoarthritis, rheumatoid arthritis, or other joint conditions, since patients might not distinguish these disorders accurately in the self-report setting.

### Statistical analysis

We characterized the longitudinal cohort of participants who received lumbar spine MRI both at baseline and 3-year follow-up. We examined associations between covariates and the two symptom outcomes, using the chi-square test for categorical variables, and Student’s t-test for continuous variables. We then used bivariable logistic regression models to examine the association between incident MRI findings and our symptom outcomes, emphasizing the primary MRI predictors of interest described in Figure 1. Where zero cell counts rendered odds ratios inestimable using ordinary logistic regression, we used exact logistic regression. Due to the low frequency of incident MRI findings, we expected *a priori* to have low power to detect statistical associations exceeding the commonly used threshold of p = 0.05. Therefore, we planned in advance to focus our interpretation of results on the magnitude of associations detected, rather than emphasizing statistical significance according to p-values. We also examined multivariable associations between incident MRI findings and symptom outcomes, adjusting for selected covariates that demonstrated at least statistical trends toward an association with the symptom outcomes (p ≤ 0.15) in the earlier bivariable models; depression was forced into the models based on its importance as a risk factor for pain [14,24]. In the last stage of the analysis, we examined whether the associations between incident MRI findings and symptom outcomes varied by outcome. To do this, we used bivariate logistic regression models with the sandwich variance estimator to model the chronic LBP and radicular symptom outcomes simultaneously, while accounting for clustering of these two outcomes on the level of the individual participant [25]. We expected that MRI findings with a putative link to low back pain would have large magnitude positive associations with low back pain, but associations with radicular symptoms close to unity (odds ratio [OR] = 1.0). Conversely, we expected that MRI findings with a putative link to radicular symptoms would have large magnitude positive associations with radicular symptoms, but associations with low back pain close to unity. All data analyses were performed with Stata software version 11.2. [26].

## Results

Of the 148 participants who met study criteria, 123 returned for a follow-up MRI after 3 years and comprised the longitudinal MRI cohort. These participants contributed symptom outcome data at 91% of follow-up timepoints [14]. Those in the longitudinal cohort were similar to those who did not return for a follow-up MRI with respect to all covariates (data not shown), except for being less likely to report having arthritis (19% vs. 44%; p = 0.007).

Table 1 presents characteristics of the longitudinal cohort. The average age was 53.4 years, and the majority of participants were white males. Over 3 years of follow-up, 20% of participants reported incident chronic LBP, and 57% reported incident radicular symptoms. The 3-year incidence of lumbar spine MRI findings ranged from 1.6% (2 new cases) for moderate/severe central canal stenosis to 8.9% for disc dessication (11 new cases).

**Table 1:** Characteristics of the longitudinal cohort (n = 123)

	**Mean (Standard deviation) or N (%)**
**Sociodemographics**	
Age (yrs.)	53.4 (9.4)
Female sex	16 (13.0%)
Race	
White	104 (84.6%)
Black	10 (8.1%)
Other	9 (7.3%)
**Clinical characteristics at baseline**	
Body mass index (kg/m^2^)	28.4 (5.2)
Current smoking	26 (21.1%)
Depression	19 (15.6%)
Arthritis	23 (19.0%)
Prior low back pain	55 (44.7%)
Prior low back pain with sciatica	11 (8.9%)
**Incident MRI findings over 3-year follow-up**	
Modic changes (any)	10 (8.1%)
Modic changes (type I)	7 (5.7%)
Facet joint hypertrophy	9 (7.3%)
Annular fissures	6 (4.9%)
Disc height narrowing	6 (4.9%)
Disc dessication	11 (8.9%)
Disc bulging	6 (4.9%)
Spondylolisthesis	4 (3.3%)
Central stenosis	2 (1.6%)
Disc extrusions	5 (4.1%)
Nerve root impingement	4 (3.3%)
Lateral recess stenosis	9 (7.3%)
Disc protrusion	9 (7.3%)
**Incident symptoms over 3-year follow-up**	
Chronic low back pain	24 (19.5%)
Radicular symptoms	70 (56.9%)

In bivariable analyses, individuals with incident chronic LBP were significantly more likely to report having arthritis at study inception (33.3% vs. 15.5%, p = 0.046) than those without chronic LBP, but were otherwise similar with respect to the covariates examined (data not shown). Individuals with incident radicular symptoms were more likely to have depression at study inception (21.4% vs. 7.7%; p = 0.039) than those without radicular symptoms, and showed a trend towards being more likely to have had a prior history of sciatica (12.9% vs. 3.8%; p = 0.11), but were otherwise comparable with respect to covariates examined (data not shown).

Table 2 presents bivariable analyses of associations between incident MRI findings and incident spine-related symptom outcomes. Of the primary MRI predictors, several incident MRI findings showed large magnitude associations with symptoms. Incident annular fissures conferred a greater odds of incident chronic LBP (OR 4.6 [95% CI 0.9-24.2]), however, there were still 3 cases (50%) of incident annular fissures where participants did not develop incident chronic LBP. Incident central canal stenosis, disc extrusions, and nerve root impingement occurred in 2, 5, and 4 participants respectively, and every participant with these incident MRI findings developed incident radicular symptoms. Incident disc extrusions (OR 5.4 [95% CI 0.7-∞)]) and nerve root impingement (OR 4.1 [95% CI 0.5-∞)]) each conferred a greater odds of incident radicular symptoms. Although not a primary predictor of interest, incident spondylolisthesis conferred a greater odds of incident chronic LBP (OR 8.9 [95% CI 0.8-102.7]) than any other finding. As expected due to the small number of incident MRI findings, none of the bivariable associations had *p*-values < 0.05. In multivariable analyses, we adjusted for depression and arthritis in models with a LBP outcome, and depression and prior sciatica in models with a radicular symptom outcome. These results were not materially different from the bivariable results, except for the association of incident annular fissures with LBP (OR 6.0 [95% CI 1.1-33.1]), which became stronger and statistically significant after we adjusted for the potential confounders of depression and arthritis (other data not shown). In post-hoc examination of the 3 cases that developed incident annular fissures but did not develop chronic LBP (using the LBP definition that we had specified *a priori)*, all 3 cases reported having some LBP at one or more follow-up timepoints, but in all instances LBP was of a severity less than ‘moderately’ bothersome.

**Table 2:** Associations between incident MRI findings and incident symptoms*

**MRI findings with conceptual links to chronic low back pain**				
	**Chronic low back pain (n = 24) n (%)**	**No low back pain (n = 99) n (%)**	**Odds ratio (95% confidence interval)**	***p*-value**
Primary Predictors of Interest				
**Endplate changes (any)**	2 (8.3)	8 (8.1)	1.0 (0.2-5.2)	0.97
**Endplate changes (type I)**	2 (8.3)	5 (5.1)	1.7 (0.3-9.5)	0.54
**Facet joint hypertrophy**	3 (12.5)	6 (6.1)	2.2 (0.5-9.6)	0.29
**Annular fissures**	3 (12.5)	3 (3.0)	4.6 (0.9-24.2)	0.074
Secondary Predictors of Interest				
Disc height narrowing	1 (4.2)	5 (5.1)	0.8 (0.1-7.3)	0.86
Disc dessication	3 (12.5)	8 (8.1)	1.6 (0.4-6.7)	0.50
Disc bulging	2 (8.3)	4 (4.0)	2.2 (0.4-12.5)	0.39
Spondylolisthesis	2 (8.3)	1 (1.0)	8.9 (0.8-102.7)	0.080
**MRI findings with conceptual links to radicular symptoms**				
	**Radicular symptoms (n = 70) n (%)**	**Radicular symptoms (n = 53) n (%)**	**Odds ratio (95% confidence interval)**	***p*-value**
Primary Predictors of Interest				
Central canal stenosis*	2 (2.9)	0 (0.0)	1.8 (0.1-∞)	0.64
Disc extrusions*	5 (7.1)	0 (0.0)	5.4 (0.7-∞)	0.11
Nerve root impingement*	4 (5.7)	0 (0.0)	4.1 (0.5-∞)	0.20
Secondary Predictors of Interest				
Spondylolisthesis	2 (2.9)	1 (1.9)	1.5 (0.1-17.3)	0.73
Lateral recess stenosis	7 (10.0)	2 (3.8)	2.8 (0.6-14.2)	0.21
Disc protrusions	5 (7.1)	4 (7.6)	0.9 (0.2-3.7)	0.93

*Odds ratios calculated using exact logistic regression.

Table 3 presents the results of multivariable logistic regression analyses examining whether MRI finding associations with symptom outcomes (chronic LBP vs. radicular symptoms) varied according to the specific outcome involved. Only certain MRI findings showed differential associations with a magnitude and direction consistent with what was expected based on conceptual grounds (as in Figure 1). Annular fissures, facet joint hypertrophy, and disc bulging showed positive associations with LBP, but essentially null associations with radicular symptoms. Disc extrusions and nerve root impingement showed positive associations with radicular symptoms, but essentially null associations with LBP.

**Table 3:** Differences in MRI associations with symptoms, according to the symptom involved*

**MRI findings with conceptual links to chronic low back pain**			
	**Associations with low back pain OR (95% CI)**	**Associations with radicular symptoms OR (95% CI)**	***p*-value for interaction**
**Endplate changes (any)**	1.0 (0.2-5.2)	1.1 (0.3-4.3)	0.90
**Endplate changes (type I)**	1.7 (0.3-9.5)	1.0 (0.2-4.7)	0.52
**Facet joint hypertrophy**	2.2 (0.5-9.6)	0.6 (0.1-2.3)	0.12
**Annular fissures**	4.6 (0.9-24.2)	0.7 (0.1-3.9)	0.065
Disc height narrowing	0.8 (0.1-7.3)	1.5 (0.3-8.8)	0.59
Disc dessication	1.6 (0.4-6.7)	1.4 (0.4-4.9)	0.81
Disc bulging	2.2 (0.4-12.5)	0.7 (0.1-3.9)	0.13
**MRI findings with conceptual links to both chronic low back pain and radicular symptoms**			
Spondylolisthesis	8.9 (0.8-102.7)	1.5 (0.1-17.5)	0.41
Disc protrusions	1.2 (0.2-6.2)	0.9 (0.2-3.7)	0.77
**MRI findings with conceptual links to radicular symptoms**			
**Central canal stenosis**	4.2 (0.1- 338)	1.8 (0.1-∞)	NA^†^
**Disc extrusions**	1.0 (0.02-11.1)	5.4 (0.7-∞)	NA^†^
**Nerve root impingement**	1.4 (0.02-18.2)	4.1 (0.5-∞)	NA^†^
Lateral recess stenosis	6.3 (1.5-25.6)	2.8 (0.6-14.3)	0.45

*primary MRI predictors of interest in bold; ^†^p-values for interaction were not calculable due to the presence of zero cell counts.

## Discussion

In this longitudinal cohort study, the 3-year incidence of new MRI findings was quite low (range 2-8%), and consequently the majority of participants who developed incident symptoms did not concurrently develop new MRI findings. When examining associations between new MRI findings and specific spine-related outcomes, only three large magnitude effects were found for our primary MRI predictors of interest which corresponded with our expectations: the association of incident annular fissures with chronic LBP, and the association of disc extrusions and nerve root impingement with radicular symptoms. Annular fissures were not specific for incident chronic LBP, in that 3 subjects (50.0%) with new annular fissures did not develop chronic LBP. In contrast, all participants with disc extrusions and nerve root impingement developed symptoms, and these findings are therefore quite specific for the production of radicular symptoms. No other incident MRI findings showed large effects on symptoms.

There are scarce prior reports examining longitudinal associations between incident lumbar MRI findings and symptoms, and direct comparisons with our findings are complicated by the different symptom outcomes used in earlier work. Borenstein et al. reported on 31 individuals who received longitudinal lumbar MRI assessments separated by 7 years, where incident symptoms were assessed using a single questionnaire recalling symptoms over the 7 years prior [27]. Having any incident MRI finding conferred a relative risk of 3.5 for incident LBP, but no individual MRI findings were strongly linked with incident symptoms. Elfering et al. reported on 41 individuals who received longitudinal lumbar MRI assessments separated by 5 years [28]. Individuals with incident disc degeneration (disc dessication and/or height narrowing), and individuals with incident vertebral endplate changes showed absent to weak associations with LBP by various definitions, and no associations were seen between annular fissures and LBP. Jarvik et al. reported previously on the same LAIDBACK participants described in the current article, who were followed for 3 years and assessed for the association between incident MRI findings and incident symptoms, using a composite outcome of any LBP and/or radicular symptoms. They did not detect statistically significant associations (p < 0.05), but noted that all subjects with new extrusions, nerve root impingement, and central canal stenosis developed new symptoms. The current work differs from the original LAIDBACK analysis in that we applied more specific symptom definitions that separated LBP from radicular symptoms, corresponding to the distinctions made in actual clinical practice. The importance of distinguishing LBP, radicular symptoms, and composite outcomes combining these two symptoms is more than just semantic: a recent systematic review of associations between lumbar MRI findings and chronic LBP excluded the LAIDBACK cohort from consideration due to the use of this composite symptom outcome [4]. Our findings, and that of prior longitudinal studies, demonstrate that the annual incidence of new MRI findings is extremely low, rendering all published studies severely underpowered to detect statistically significant associations with incident symptom outcomes. Our study showed no large magnitude longitudinal associations between disc degeneration (height narrowing, dessication, bulging) or endplate changes and chronic LBP, which is generally consistent with prior longitudinal studies. We had not expected disc degeneration to be associated with LBP due to the modest effects seen in prior cross-sectional studies [4]. However, we had expected to see a large magnitude association between endplate changes and LBP, based on cross-sectional studies reporting positive associations with effects ranging from OR 2.0 to 6.1 [18,29]; our 95% confidence intervals do not rule out associations of this magnitude. Our finding of a large magnitude longitudinal association between annular fissures and LBP is consistent with some cross-sectional studies showing positive associations with LBP (OR 2.5-4.6 [5,18,30]), but inconsistent with one cross-sectional study suggesting a protective effect on LBP [31], and the longitudinal study by Elfering, which detected no association [28].

Earlier studies by Borenstein et. al and Jarvik et. al showed conflicting results for the association between incident disc extrusions and incident symptoms; our secondary analysis of the same cohort reported on by Jarvik et. al suggests that disc extrusions are associated with radicular symptoms, but not with LBP. Similarly, we found nerve root impingement to have a large magnitude association with radicular symptoms, but not with LBP. These findings are consistent with the common clinical reasoning that extrusions and nerve impingement primarily produce radicular symptoms due to nerve involvement, and in this setting LBP may or may not be present. These results suggest that when a new MRI demonstrates a disk extrusion or nerve root impingement that was not previously seen, in a patient with new radicular symptoms, the new MRI finding is a probable cause of radicular symptoms. However, the overwhelming majority of individuals with new radicular symptoms will not have relevant new MRI findings.

A strength of our study is the clear distinction made between the symptom outcomes of LBP and radicular symptoms. We conducted separate analyses for specific MRI findings with specific symptom outcomes, based on prespecified relationships that were thought to have greater conceptual importance. Our analyses examining how MRI finding effects varied with the symptom outcome confirmed several instances where the expected association with one symptom outcome was not seen with the other symptom outcome (i.e. the association of annular fissures with LBP but not radicular symptoms, and the association of disc extrusions/nerve impingement with radicular symptoms but not LBP). This highlights the importance of the case definitions used in back pain research. Variation in case definition, and the tendency to combine symptoms into composite outcomes in epidemiologic imaging studies, may obscure real and important biological relationships. Although we made efforts to optimize case definition, even these definitions had important limitations. For instance, our definition of radicular symptoms resulted in a 3-year prevalence of 57% for incident pain, weakness, numbness, or tingling, which is substantially higher than would be expected for sciatica. Our definition of radicular symptoms likely included not only true cases of sciatica, but other apparent ‘cases’ where limb symptoms were explained by factors unrelated to a lumbosacral radicular syndrome (i.e. limb pain due to hip/knee osteoarthritis, or occasional limb paresthesias due to transient nerve impingement during leg crossing). An interview and examination by a trained clinician during the episode of radicular symptoms may have improved case definition accuracy. Another potential source of misclassification in longitudinal studies of imaging associations with symptoms is recall bias. The potential for misclassification due to inaccurate recall is high for conditions like acute LBP and sciatica, where the natural history may involve transient episodes of severe pain that resolve quickly, but may also recur or otherwise vary over time. Our study, in contrast to earlier studies [27,28], sampled participants at regular intervals every 4 months for 3 years, and was likely to have minimized such recall inaccuracy. Nevertheless, sampling at 4-month intervals has shortcomings as well: a period of LBP lasting only 1 month could have bridged two 4-month sampling periods and met criteria for LBP using our case definition, while not exceeding the usual 3-month or 6-month threshold used for defining persistent LBP.

Certain other limitations of our study warrant further mention. First, the low 3-year incidence of MRI findings limited the statistical stability of our point estimates, and future longitudinal MRI studies will require a longer duration of follow-up. The low incidence of new MRI findings also limited our ability to simultaneously adjust for multiple incident MRI findings in the same multivariable models; for a multifactorial condition such as LBP, this might obscure important relationships. Second, our analysis made multiple statistical comparisons, raising the concern that identified associations may represent type I error and not reflect a true biologic effect. This is of particular concern for the association of annular fissures with LBP, where prior association studies have had mixed results [5,18,30,31]. Although we attempted to limit multiple comparisons by prioritizing seven ‘primary predictors’ where associations were expected, and relegating other factors to secondary status, longitudinal studies are needed to replicate our findings. Third, our study ascertained MRI findings at only two timepoints. Since specific MRI findings are known to improve over time, particularly with disc extrusions (which often resorb [32,33]), the lack of an interim imaging timepoint may have created misclassification by failing to detect some MRI findings which developed at the precise time of symptom onset, but had improved by the 3-year follow-up MRI. Alternatively, new MRI findings on the 3-year follow-up MRI may have developed *after* new symptoms had occurred. An ideal design for future longitudinal MRI studies would include pre- and post- MRI assessments separated by ≥ 6 years, with the addition of an interim MRI assessment at the time of incident symptoms, and/or at an interim control period. Fourth, although interobserver variability between the two neuroradiologist readers were calculated using the baseline MRIs, interobserver variability for *changes* in MRI findings (i.e. incident findings) was not assessed. Therefore the reliability estimates for MRI interpretation that we estimated may not apply to scans that demonstrated incident findings. Fifth, the use of dichotomies to define the abnormality itself could introduce inaccuracy, since there is a dearth of prior studies examining what thresholds for each of these MRI findings best correlate with symptoms, and what level of reliability pertains to each threshold. Lastly, the current study includes the same participants that were described in an earlier report [14]. This analysis, however, was distinct from that performed previously in that different (and more specific) symptom outcomes were used, with a focus entirely on associations between longitudinal changes in MRI findings and incident symptoms. As described above, the symptom outcome definitions for chronic LBP and radicular symptoms were specified prior to conducting the analyses described here.

Although our findings did not detect multiple large magnitude and statistically significant associations between incident MRI findings and incident LBP or sciatica, they should not be taken to suggest that research studies of imaging for LBP are not a vital area for future research. Imaging research into the structural correlates of LBP is an essential step towards delineating that component of the LBP experience that is explained by structural or anatomic factors, understanding that these biological correlates of pain are only one part of a complex process most accurately viewed within the context of a biopsychosocial framework [34]. Currently there is no evidence that imaging improves LBP outcomes in standard clinical practice; however, without ongoing back pain imaging research, there will be no opportunity for imaging methods and approaches to improve to the point where they can refine clinical decision-making. Instead, we emphasize the need for future back pain imaging research, while taking care to distinguish between what is of value from a research perspective, and what is ready for translation into actual clinical practice. Given the low specificity of most MRI findings in both prevalence and incidence studies, we suspect that the time of translation into clinical practice is still far off in the future.

## Conclusions

In conclusion, this study demonstrates that even when applying more specific definitions for spine-related symptom outcomes, few MRI findings show large magnitude associations with symptom outcomes. The longitudinal association of annular fissures with LBP, and the association of disc extrusions and nerve root impingement with radicular symptoms, require confirmation in studies with comparable longitudinal designs. Nevertheless, the 3-year incidence of these findings is very low, and at best explains only a very small proportion of incident symptomatic cases.

## Abbreviations

MRI: Magnetic resonance imaging; LBP: Low back pain; VA: Veterans affairs; LAIDBACK: Longitudinal assessment of imaging and disability of the back; SACQ: Self-administered comorbidity questionnaire; OR: Odds ratio; CI: Confidence interval.

## Competing interests

The authors declare that they have no competing interests relevant to the content of this manuscript, except for the following: Dr. Jarvik is a member of the GE Healthcare Advisory Board for Comparative Effectiveness and a consultant for HealthHelp, a Radiology Benefits Management company. He is a faculty member at the Radiological Society of North America Clinical Trials Workshop.

## Authors’ contributions

PS was involved with study concept and design, analysis of data, interpretation of data, and drafting of the manuscript. EB was involved with study design and interpretation of data. JG was involved with interpretation of data. CF was involved with analysis of data. JGJ was involved with study design and interpretation of data. All authors were involved with critical revision of the manuscript for important intellectual content and approved the final version of the manuscript.

## Pre-publication history

The pre-publication history for this paper can be accessed here:

http://www.biomedcentral.com/1471-2474/15/152/prepub

## Supplementary Material

Additional file 1 MRI Imaging measures. Click here for file
