# Supplementary material for: Longitudinal associations between incident lumbar spine MRI findings and chronic low back pain or radicular symptoms: retrospective analysis of data from the longitudinal assessment of imaging and disability of the back (LAIDBACK)
Source: BMC Musculoskelet Disord. 2014 May 13;15:152. doi: 10.1186/1471-2474-15-152 (PMC4024651; doi:10.1186/1471-2474-15-152)
Supplement: Additional file 1 — MRI Imaging measures. [file 1471-2474-15-152-S1.docx]

**Additional file: MRI IMAGING MEASURES**

| **MRI Finding** | **Definition** | **Threshold for incident change at any spinal level** |
| --- | --- | --- |
| **Endplate changes**[[1](#_ENREF_1)] |  | None vs. any endplate changes |
| Type I | Low T1 and high T2 (endplate edema) | None vs. type I endplate change |
| Type II | High T1 and low to intermediate T2 (fatty change) |  |
| Type III | Low T1 and low T2 (sclerosis) |  |
| **Facet hypertrophy** |  |  |
| Mild | Mild hypertrophy with minimal or no neural foramen encroachment |  |
| Moderate | Moderate hypertrophy with moderate neural foramen or canal  encroachment | None/mild vs.  moderate/severe |
| Severe | Extensive hypertrophy with severe neural foramen or canal encroachment |  |
| **Annular fissure** [[2](#_ENREF_2)] | Focal hyperintensity of the annulus on T2-weighted image. | None vs. any fissure |
| **Disc height narrowing** [[3](#_ENREF_3)] | Decrease in disc height compared with expected height of a hydrated disc at the same level | None vs. any narrowing |
| **Disc dessication** |  |  |
| Mild | Mild diffuse decrease in signal or focal moderate or severe decrease <1⁄3 of disc area on sagittal image. |  |
| Moderate | Moderate diffuse decrease in signal or focal moderate or severe decrease 1⁄3–2⁄3 of disc area on sagittal image. | None/mild vs.  moderate/severe |
| Severe | Severe diffuse decrease (no residual high T2 signal) >2⁄3 of disc area on sagittal image. |  |
| **Disk bulging** | Circumferential symmetric extension of the disc beyond the interspace | None vs. any bulging |
| **Spondylolisthesis** | Displacement of one vertebra atop another in the sagittal plane | Any new or worsened spondylolisthesis |
| **Disc protrusion** [[4](#_ENREF_4)] | Focal or asymmetric extension of the disc beyond the interspace with the base against the disc of origin broader than any other dimension of the protrusion. | None vs. any protrusion |
| **Central canal stenosis** |  |  |
| Mild | Encroachment on central canal but abundant cerebrospinal fluid still present around roots |  |
| Moderate | Crowded roots with only small amount of residual cerebrospinal fluid | None/mild vs.  moderate/severe |
| Severe | No residual cerebrospinal fluid |  |
| **Disc extrusions** [[4](#_ENREF_4)] | More extreme extension of the disk of origin narrower than the diameter of the extruding material itself or with no connection between the material and the disk | None vs. any extrusion |
| **Nerve root**  **Impingement** [[5](#_ENREF_5)] | Contact/displacement/compression of nerve root and or thecal sac | None vs. any impingement |
| **Lateral recess stenosis** | Narrowing of the lateral recess graded subjectively as normal, mild, moderate, or severe | None vs. any |

1. Modic MT, Masaryk TJ, Ross JS, Carter JR: **Imaging of degenerative disk disease**. *Radiology* 1988, **168**(1):177-186.

2. Aprill C, Bogduk N: **High-intensity zone: a diagnostic sign of painful lumbar disc on magnetic resonance imaging**. *Br J Radiol* 1992, **65**(773):361-369.

3. Dabbs VM, Dabbs LG: **Correlation between disc height narrowing and low-back pain**. *Spine (Phila Pa 1976)* 1990, **15**(12):1366-1369.

4. Jensen MC, Brant-Zawadzki MN, Obuchowski N, Modic MT, Malkasian D, Ross JS: **Magnetic resonance imaging of the lumbar spine in people without back pain**. *N Engl J Med* 1994, **331**(2):69-73.

5. Weishaupt D, Zanetti M, Hodler J, Boos N: **MR imaging of the lumbar spine: prevalence of intervertebral disk extrusion and sequestration, nerve root compression, end plate abnormalities, and osteoarthritis of the facet joints in asymptomatic volunteers**. *Radiology* 1998, **209**(3):661-666.
